# 4-[(4-Methyl­phen­yl)amino]­pent-3-en-2-one

**DOI:** 10.1107/S1600536810043680

**Published:** 2010-10-31

**Authors:** Gertruida J. S. Venter, Gideon Steyl, Andreas Roodt

**Affiliations:** a Department of Chemistry, University of the Free State, PO Box 339, Bloemfontein 9300, South Africa

## Abstract

The title enamino­ketone, C_12_H_15_NO, is a derivative of 4-(phenyl­amino)­pent-3-en-2-one with an approximately planar pentenone backbone, the greatest displacement from the plane being 0.042 (1) Å; the asymmetry in C—C distances in the group suggests the presence of unsaturated bonds. The dihedral angle between the benzene ring and the pentenone plane is 29.90 (4)°. In the crystal, an intra­molecular N—H⋯O inter­action and an inter­molecular C—H⋯O hydrogen bond are observed.

## Related literature

For synthetic background, see: Shaheen *et al.* (2006 ▶); Venter *et al.* (2010 ▶). For applications of enamino­ketones, see: Brink *et al.* (2010 ▶); Chen & Rhodes (1996 ▶); Pyżuk *et al.* (1993 ▶); Roodt & Steyn (2000 ▶); Tan *et al.* (2008 ▶); Xia *et al.* (2008 ▶). For structures of related ligand systems, see: Venter *et al.* (2009*a* ▶,*b* ▶).
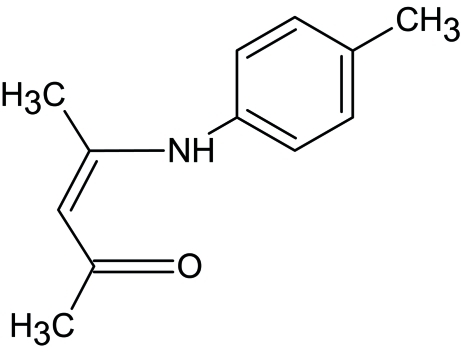


## Experimental

### 

#### Crystal data

C_12_H_15_NO
*M* *_r_* = 189.25
Monoclinic, 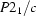

*a* = 10.0736 (2) Å
*b* = 10.8800 (2) Å
*c* = 10.0723 (2) Å
β = 110.291 (1)°
*V* = 1035.43 (3) Å^3^
*Z* = 4
Mo *K*α radiation
μ = 0.08 mm^−1^
*T* = 100 K
0.34 × 0.31 × 0.2 mm

#### Data collection

Bruker X8 APEXII 4K Kappa CCD diffractometer
Absorption correction: multi-scan (*SADABS*; Bruker, 2004 ▶) *T*_min_ = 0.974, *T*_max_ = 0.985
17880 measured reflections
2257 independent reflections
2011 reflections with *I* > 2σ(*I*)
*R*_int_ = 0.024

#### Refinement

*R*[*F* ^2^ > 2σ(*F* ^2^)] = 0.035
*wR*(*F* ^2^) = 0.099
*S* = 1.05
2257 reflections
134 parameters
H atoms treated by a mixture of independent and constrained refinement
Δρ_max_ = 0.28 e Å^−3^
Δρ_min_ = −0.20 e Å^−3^

### 

Data collection: *APEX2* (Bruker, 2005 ▶); cell refinement: *SAINT-Plus* (Bruker, 2004 ▶); data reduction: *SAINT-Plus*; program(s) used to solve structure: *SHELXS97* (Sheldrick, 2008 ▶); program(s) used to refine structure: *SHELXL97* (Sheldrick, 2008 ▶); molecular graphics: *DIAMOND* (Brandenburg & Putz, 2005 ▶); software used to prepare material for publication: *WinGX* (Farrugia, 1999 ▶).

## Supplementary Material

Crystal structure: contains datablocks global, I. DOI: 10.1107/S1600536810043680/is2620sup1.cif

Structure factors: contains datablocks I. DOI: 10.1107/S1600536810043680/is2620Isup2.hkl

Additional supplementary materials:  crystallographic information; 3D view; checkCIF report

## Figures and Tables

**Table 1:** Hydrogen-bond geometry (Å, °)

*D*—H⋯*A*	*D*—H	H⋯*A*	*D*⋯*A*	*D*—H⋯*A*
C116—H116⋯O12^i^	0.95	2.55	3.462 (1)	160
N11—H11⋯O12	0.916 (16)	1.859 (16)	2.6463 (13)	142.7 (15)

Symmetry code: (i) 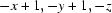.

**Table 2:** Comparative geometric parameters (Å, °) for free and coordinated *N*,*O*-bidendate (*N*,*O*-bid) compounds

Parameter	I	II	III	IV
N11—C111	1.417 (2)	1.422 (2)	1.521 (4)/1.463 (3)	1.440 (4)
N11—C2	1.348 (1)	1.345 (2)	1.320 (4)	1.319 (4)
O12—C4	1.253 (1)	1.257 (2)	1.290 (3)	1.291 (4)
C2—C3	1.384 (2)	1.383 (3)	1.410 (4)	1.423 (4)
C3—C4	1.424 (2)	1.420 (2)	1.365 (3)	1.382 (3)
N11⋯O12	2.646 (1)	2.635 (2)	2.885 (3)	2.886 (3)
N11—C2—C4—O12	1.70 (9)	−0.5 (1)	4.1 (2)	−2.6 (2)
Dihedral angle	29.90 (3)	49.53 (5)	87.47 (4)/89.36 (8)	85.58 (8)

(I) This work. (II) Uncoordinated 4-(2-methyl­phenyl­amino)­pent-3-en-2-one (Venter *et al.*, 2010 ▶). (III) *N*,*O*-bid = 4-(2,3-dimethyl­phenyl­amino)­pent-3-en-2-onato (Venter *et al.*, 2009*a* ▶). (IV) *N*,*O*-bid = 4-(2,6-dimethyl­phenyl­amino)­pent-3-en-2-onato (Venter *et al.*, 2009*b* ▶). The dihedral angle is defined as the torsion angle between the N—C—C—C—O plane and the phenyl ring.

**Fig. 1.**
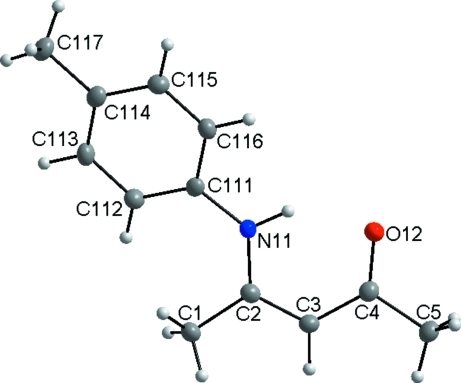
Molecular structure of the title compound. Displacement ellipsoids are drawn at the 50% probability displacement level.

## supplementary crystallographic information

### Comment

A well known system in organometallic chemistry is the β-diketone compound AcacH (acetylacetone; or when coordinated acetylacetonato, acac^-^). A multitude of derivatives have been synthesized to date, with enaminoketones being one type. Since enaminoketones contain N and O atoms as well as an unsaturated C—C bond, these electron-rich compounds are of interest in various fields including liquid crystals (Pyżuk *et al.*, 1993), fluorescence studies (Xia *et al.*, 2008) as well as formation of complexes of medical interest (Tan *et al.*, 2008; Chen & Rhodes, 1996). It also has significant application possibilities in catalysis (Roodt & Steyn, 2000; Brink *et al.*, 2010).

The title compound (Fig. 1) crystallizes in the monoclinic space group *P*2_1_/*c* with *Z* = 4. This enaminoketone is a derivative of 4-(phenylamino)pent-3-en-2-one (PhonyH; Shaheen *et al.*, 2006). Bond distances differ significantly from compounds coordinated to rhodium (Table 2; Venter *et al.*, 2009*a*,*b*), such as the distance, N1···O1, greatly increasing (~0.2 Å) upon coordination to the metal. Similarities in bond parameters to other uncoordinated enaminoketone compounds (Venter *et al.*, 2010) were observed. The C2—C3 distance of 1.385 (2) Å, *versus* the C3—C4 bond distance of 1.424 (2) Å indicates an unsaturated bond in the pentenone backbone. The dihedral angle between the benzene ring and the pentenone plane is 29.90 (4)°.

### Experimental

A solution of acetylacetone (11.02 g, 0.1101 mol), 4-Me-aniline (10.83 g, 0.1010 mol) and 2 drops of H_2_SO_4_ (conc.) in 150 ml benzene was refluxed for 24 h in a Dean–Stark trap, filtered and left to crystallize. Crystals suitable for X-ray diffraction were obtained in 16.95 g (88.67%) yield. This compound is stable in air and light over a period of several months.

### Refinement

The methyl and aromatic H atoms were placed in geometrically idealized positions and constrained to ride on their parent atoms, with C—H = 0.95 and 0.98 Å and *U*_iso_(H) = 1.5*U*_eq_(C) and 1.2*U*_eq_(C), respectively. The methyl groups were generated to fit the difference electron density and the groups were then refined as rigid rotors. The highest residual electron-density peak is 0.74 Å from C5.

### Crystal data

**Table:**

C_12_H_15_NO	*F*(000) = 408
*M**_r_* = 189.25	*D*_x_ = 1.214 Mg m^−^^3^
Monoclinic, *P*2_1_/*c*	Mo *K*α radiation, λ = 0.71073 Å
Hall symbol: -P 2ybc	Cell parameters from 8475 reflections
*a* = 10.0736 (2) Å	θ = 2.9–28.3°
*b* = 10.8800 (2) Å	µ = 0.08 mm^−^^1^
*c* = 10.0723 (2) Å	*T* = 100 K
β = 110.291 (1)°	Cuboid, white
*V* = 1035.43 (3) Å^3^	0.34 × 0.31 × 0.2 mm
*Z* = 4	

### Data collection

**Table:**

Bruker X8 APEXII 4K Kappa CCD diffractometer	2257 independent reflections
Radiation source: fine-focus sealed tube	2011 reflections with *I* > 2σ(*I*)
graphite	*R*_int_ = 0.024
ω and φ scans	θ_max_ = 27°, θ_min_ = 1.6°
Absorption correction: multi-scan (*SADABS*; Bruker, 2004)	*h* = −9→12
*T*_min_ = 0.974, *T*_max_ = 0.985	*k* = −13→13
17880 measured reflections	*l* = −12→12

### Refinement

**Table:**

Refinement on *F*^2^	Primary atom site location: structure-invariant direct methods
Least-squares matrix: full	Secondary atom site location: difference Fourier map
*R*[*F*^2^ > 2σ(*F*^2^)] = 0.035	Hydrogen site location: inferred from neighbouring sites
*wR*(*F*^2^) = 0.099	H atoms treated by a mixture of independent and constrained refinement
*S* = 1.05	*w* = 1/[σ^2^(*F*_o_^2^) + (0.0527*P*)^2^ + 0.3154*P*] where *P* = (*F*_o_^2^ + 2*F*_c_^2^)/3
2257 reflections	(Δ/σ)_max_ = 0.001
134 parameters	Δρ_max_ = 0.28 e Å^−^^3^
0 restraints	Δρ_min_ = −0.19 e Å^−^^3^

### Special details

**Table:**

Experimental. The intensity data was collected on a Bruker X8 ApexII 4 K Kappa CCD diffractometer using an exposure time of 30 s/frame. A total of 688 frames were collected with a frame width of 0.5° covering up to θ = 27.00° with 99.9% completeness accomplished.
Geometry. All e.s.d.'s (except the e.s.d. in the dihedral angle between two l.s. planes) are estimated using the full covariance matrix. The cell e.s.d.'s are taken into account individually in the estimation of e.s.d.'s in distances, angles and torsion angles; correlations between e.s.d.'s in cell parameters are only used when they are defined by crystal symmetry. An approximate (isotropic) treatment of cell e.s.d.'s is used for estimating e.s.d.'s involving l.s. planes.

### Fractional atomic coordinates and isotropic or equivalent isotropic displacement parameters (Å^2^)

**Table:**

	*x*	*y*	*z*	*U*_iso_*/*U*_eq_	
H11	0.4680 (15)	0.6492 (15)	0.1245 (16)	0.035 (4)*	
C1	0.40618 (11)	0.93113 (10)	0.19271 (12)	0.0217 (2)	
H1A	0.3029	0.923	0.1547	0.033*	
H1B	0.4343	1.0054	0.1542	0.033*	
H1C	0.4381	0.9371	0.2961	0.033*	
C2	0.47215 (10)	0.82105 (9)	0.15165 (10)	0.0172 (2)	
C3	0.58328 (10)	0.83668 (10)	0.10224 (11)	0.0184 (2)	
H3	0.6177	0.9177	0.1004	0.022*	
C4	0.64936 (10)	0.73921 (10)	0.05403 (11)	0.0189 (2)	
C5	0.76886 (11)	0.77128 (11)	0.00155 (12)	0.0237 (2)	
H5A	0.8532	0.7236	0.0543	0.036*	
H5B	0.7901	0.8592	0.0158	0.036*	
H5C	0.7407	0.7518	−0.0995	0.036*	
C111	0.30726 (10)	0.66767 (9)	0.19824 (11)	0.0168 (2)	
C112	0.26693 (11)	0.72321 (10)	0.30296 (11)	0.0199 (2)	
H112	0.3174	0.7925	0.3529	0.024*	
C113	0.15224 (11)	0.67660 (10)	0.33410 (11)	0.0213 (2)	
H113	0.1239	0.7164	0.4039	0.026*	
C114	0.07814 (10)	0.57364 (10)	0.26603 (11)	0.0197 (2)	
C115	0.12396 (11)	0.51612 (10)	0.16576 (11)	0.0206 (2)	
H115	0.0773	0.4437	0.1203	0.025*	
C116	0.23603 (11)	0.56237 (10)	0.13106 (11)	0.0194 (2)	
H116	0.2644	0.5223	0.0614	0.023*	
C117	−0.04840 (11)	0.52533 (11)	0.29731 (13)	0.0257 (3)	
H11A	−0.0518	0.5634	0.3843	0.039*	
H11B	−0.0404	0.436	0.3095	0.039*	
H11C	−0.1351	0.5453	0.2184	0.039*	
N11	0.42309 (9)	0.70705 (8)	0.15977 (9)	0.0176 (2)	
O12	0.61277 (8)	0.62890 (7)	0.05166 (8)	0.0227 (2)	

### Atomic displacement parameters (Å^2^)

**Table:**

	*U*^11^	*U*^22^	*U*^33^	*U*^12^	*U*^13^	*U*^23^
C1	0.0245 (5)	0.0165 (5)	0.0269 (5)	0.0011 (4)	0.0124 (4)	−0.0005 (4)
C2	0.0182 (5)	0.0165 (5)	0.0155 (5)	0.0009 (4)	0.0042 (4)	0.0003 (4)
C3	0.0191 (5)	0.0164 (5)	0.0196 (5)	−0.0013 (4)	0.0068 (4)	0.0002 (4)
C4	0.0174 (5)	0.0206 (5)	0.0185 (5)	0.0000 (4)	0.0059 (4)	0.0013 (4)
C5	0.0212 (5)	0.0240 (6)	0.0292 (6)	−0.0016 (4)	0.0127 (4)	0.0002 (4)
C111	0.0156 (4)	0.0167 (5)	0.0182 (5)	0.0028 (4)	0.0061 (4)	0.0035 (4)
C112	0.0216 (5)	0.0191 (5)	0.0191 (5)	−0.0010 (4)	0.0073 (4)	−0.0016 (4)
C113	0.0224 (5)	0.0238 (6)	0.0207 (5)	0.0029 (4)	0.0114 (4)	0.0000 (4)
C114	0.0162 (5)	0.0215 (5)	0.0216 (5)	0.0032 (4)	0.0069 (4)	0.0048 (4)
C115	0.0193 (5)	0.0188 (5)	0.0229 (5)	−0.0012 (4)	0.0062 (4)	−0.0006 (4)
C116	0.0205 (5)	0.0189 (5)	0.0200 (5)	0.0024 (4)	0.0087 (4)	−0.0005 (4)
C117	0.0200 (5)	0.0289 (6)	0.0313 (6)	0.0002 (4)	0.0129 (4)	0.0035 (5)
N11	0.0182 (4)	0.0156 (4)	0.0214 (4)	0.0016 (3)	0.0100 (3)	0.0002 (3)
O12	0.0245 (4)	0.0171 (4)	0.0306 (4)	0.0000 (3)	0.0145 (3)	−0.0006 (3)

### Geometric parameters (Å, °)

**Table:**

C1—C2	1.4959 (14)	C111—N11	1.4173 (13)
C1—H1A	0.98	C112—C113	1.3934 (14)
C1—H1B	0.98	C112—H112	0.95
C1—H1C	0.98	C113—C114	1.3882 (16)
C2—N11	1.3482 (13)	C113—H113	0.95
C2—C3	1.3842 (14)	C114—C115	1.3957 (15)
C3—C4	1.4240 (15)	C114—C117	1.5097 (14)
C3—H3	0.95	C115—C116	1.3870 (14)
C4—O12	1.2533 (13)	C115—H115	0.95
C4—C5	1.5138 (14)	C116—H116	0.95
C5—H5A	0.98	C117—H11A	0.98
C5—H5B	0.98	C117—H11B	0.98
C5—H5C	0.98	C117—H11C	0.98
C111—C112	1.3929 (14)	N11—H11	0.916 (16)
C111—C116	1.3958 (15)		
			
C2—C1—H1A	109.5	C111—C112—C113	119.66 (10)
C2—C1—H1B	109.5	C111—C112—H112	120.2
H1A—C1—H1B	109.5	C113—C112—H112	120.2
C2—C1—H1C	109.5	C114—C113—C112	121.89 (10)
H1A—C1—H1C	109.5	C114—C113—H113	119.1
H1B—C1—H1C	109.5	C112—C113—H113	119.1
N11—C2—C3	119.61 (9)	C113—C114—C115	117.59 (9)
N11—C2—C1	120.83 (9)	C113—C114—C117	121.75 (10)
C3—C2—C1	119.56 (9)	C115—C114—C117	120.66 (10)
C2—C3—C4	124.27 (10)	C116—C115—C114	121.46 (10)
C2—C3—H3	117.9	C116—C115—H115	119.3
C4—C3—H3	117.9	C114—C115—H115	119.3
O12—C4—C3	123.40 (9)	C115—C116—C111	120.14 (10)
O12—C4—C5	118.58 (9)	C115—C116—H116	119.9
C3—C4—C5	118.02 (9)	C111—C116—H116	119.9
C4—C5—H5A	109.5	C114—C117—H11A	109.5
C4—C5—H5B	109.5	C114—C117—H11B	109.5
H5A—C5—H5B	109.5	H11A—C117—H11B	109.5
C4—C5—H5C	109.5	C114—C117—H11C	109.5
H5A—C5—H5C	109.5	H11A—C117—H11C	109.5
H5B—C5—H5C	109.5	H11B—C117—H11C	109.5
C112—C111—C116	119.16 (9)	C2—N11—C111	130.44 (9)
C112—C111—N11	124.05 (9)	C2—N11—H11	111.6 (9)
C116—C111—N11	116.69 (9)	C111—N11—H11	117.5 (9)
			
N11—C2—C3—C4	1.92 (15)	C113—C114—C115—C116	−2.38 (16)
C1—C2—C3—C4	−176.91 (9)	C117—C114—C115—C116	177.08 (10)
C2—C3—C4—O12	0.03 (16)	C114—C115—C116—C111	0.96 (16)
C2—C3—C4—C5	179.34 (9)	C112—C111—C116—C115	1.83 (15)
C116—C111—C112—C113	−3.10 (15)	N11—C111—C116—C115	178.26 (9)
N11—C111—C112—C113	−179.26 (9)	C3—C2—N11—C111	−175.93 (9)
C111—C112—C113—C114	1.67 (16)	C1—C2—N11—C111	2.88 (16)
C112—C113—C114—C115	1.06 (16)	C112—C111—N11—C2	−36.50 (16)
C112—C113—C114—C117	−178.39 (10)	C116—C111—N11—C2	147.26 (10)

### Hydrogen-bond geometry (Å, °)

**Table:**

*D*—H···*A*	*D*—H	H···*A*	*D*···*A*	*D*—H···*A*
C116—H116···O12^i^	0.95	2.55	3.462 (1)	160
N11—H11···O12	0.916 (16)	1.859 (16)	2.6463 (13)	142.7 (15)

Symmetry codes: (i) −*x*+1, −*y*+1, −*z*.

### Table 2 Comparative geometric parameters (Å, °) for free and coordinated N,O-bidendate (N,O-bid) compounds

**Table:**

Parameter	I	II	III	IV
N11—C111	1.417 (2)	1.422 (2)	1.521 (4)/1.463 (3)	1.440 (4)
N11—C2	1.348 (1)	1.345 (2)	1.320 (4)	1.319 (4)
O12—C4	1.253 (1)	1.257 (2)	1.290 (3)	1.291 (4)
C2—C3	1.384 (2)	1.383 (3)	1.410 (4)	1.423 (4)
C3—C4	1.424 (2)	1.420 (2)	1.365 (3)	1.382 (3)
N11···O12	2.646 (1)	2.635 (2)	2.885 (3)	2.886 (3)
N11—C2—C4—O12	1.70 (9)	-0.5 (1)	4.1 (2)	-2.6 (2)
Dihedral angle	29.90 (3)	49.53 (5)	87.47 (4)/89.36 (8)	85.58 (8)

(I) This work. (II) Uncoordinated 4-(2-methylphenylamino)pent-3-en-2-onato (Venter *et al.*, 2010). (III) N,O-bid = 4-(2,3-dimethylphenylamino)pent-3-en-2-onato (Venter *et al.*, 2009*a*). (IV) N,O-bid = 4-(2,6-dimethylphenylamino)pent-3-en-2-onato (Venter *et al.*, 2009*b*). The dihedral angle is defined as the torsion angle between the N—C—C—C—O plane and the phenyl ring. A positive angle denotes a clockwise rotation.
